# Qualification of Targeted Attribute Quantitation and New Peak Detection for Automated Multi-Attribute Method Workflows Using Liquid Handling Systems

**DOI:** 10.3390/ph19101605

**Published:** 2026-10-09

**Authors:** Kelvin Chang, Qinjingwen Cao, Chuntian Cai, Wayman Chan, Zhiqi Hao, Feng Yang

**Affiliations:** 1 Analytical Development US, Genentech, 1 DNA Way, South San Francisco, CA 94080, USA; chang.kelvin@gene.com (K.C.);; 2 Analytical Operations US, Genentech, 1 DNA Way, South San Francisco, CA 94080, USA

**Keywords:** multi-attribute method, targeted attribute quantitation, new peak detection, automated sample preparation, liquid handling systems, quality control, peptide mapping, mass spectrometry

## Abstract

**Background/Objectives:** The Multi-Attribute Method (MAM) uses LC-MS peptide mapping for targeted attribute quantitation (TAQ) and untargeted New Peak Detection (NPD) in product quality monitoring and quality control of biotherapeutics. As automated liquid handling systems are adopted, data demonstrating similar performance for both TAQ and NPD are essential to implement an automatic sample preparation workflow to fully achieve MAM capabilities. This study compares manual versus automated sample preparation workflows for both TAQ and NPD. **Methods:** Peptide mapping sample preparation was performed manually and on an automated liquid handling system. TAQ was evaluated using a reference monoclonal antibody (mAb1) while NPD was assessed by spiking a second monoclonal antibody (mAb2) into mAb1 (0.5–2% *w*/*w* relative abundance). NPD specificity and sensitivity were qualified using a product-specific peptide library, multiple bracketing comparisons, and a statistically derived Fold Change Detection (FCD) threshold. **Results:** For TAQ, the automated workflow demonstrated comparable performance to the manual method, with minimal quantitative differences and high reproducibility across 28 monitored product quality attributes. For NPD, all expected mAb2 peptides were detected with no false positives at each spike level. No new peaks were observed when comparing bracketing unspiked mAb1 samples, confirming specificity for NPD. **Conclusions:** The results demonstrate that automated sample preparation yields consistent TAQ and NPD data compared to manual methods and can be used for routine product quality monitoring.

## 1. Introduction

The multi-attribute method (MAM) based on liquid chromatography-mass spectrometry (LC-MS) peptide mapping has emerged as a powerful, consolidated approach for product quality monitoring and quality control (QC) of therapeutic monoclonal antibodies (mAbs) [1,2,3,4,5]. Aligning with Quality by Design (QbD) principles, MAM directly monitors biologically relevant attributes by providing site-specific information [1,3,4,5,6,7]. The MAM workflow has two key components: targeted attribute quantitation (TAQ) and untargeted new peak detection (NPD). While TAQ is designed to simultaneously quantify known product quality attributes (PQAs) such as post-translational modifications, NPD functions as an untargeted differential analysis limit test [1,3,5,8,9]. By comparing test samples against a well-characterized reference standard, NPD flags unexpected process-related impurities, sequence variants, and changes to the product quality profile [4,9,10,11,12].

Despite the analytical capabilities of MAM, the widespread adoption of both TAQ and NPD components in routine development and QC environments is bottlenecked by conventional manual sample preparation. Furthermore, implementing NPD presents unique challenges in balancing high sensitivity with low false-positive rates, managing sample preparation artifacts, and meeting regulatory compliance [5]. Conventional manual peptide mapping, which often involves denaturation, reduction, alkylation, and enzymatic digestion, is highly labor-intensive, time-consuming, and prone to significant analyst and laboratory variations, as well as artificial modifications if not properly designed [13,14,15,16,17,18]. To overcome these barriers and ensure high-throughput reproducibility for routine process monitoring, the industry has increasingly turned to automated liquid handling systems (such as Tecan and Hamilton platforms). Previous studies have successfully demonstrated that automating the sample preparation process minimizes day-to-day variations, improves throughput, and achieves comparable TAQ performance to manual procedures [6,15,18,19,20,21,22].

However, the primary hurdle for successful NPD implementation remains balancing high detection sensitivity with a low false-positive rate. False positives frequently arise from inherent variations in MS data acquisition, baseline noise, or artifacts introduced during sample preparation. To reduce false positives, NPD workflows often rely on aggressive software filtering criteria; unfortunately, this arbitrary thresholding can elevate the limit of detection and increase the risk of missing true unexpected impurities (false negatives) [9,11,23,24,25]. Recent interlaboratory studies, including those by the MAM consortium, emphasize that the success of NPD relies heavily on the rational, statistical design of software thresholds, such as empirically derived Fold Change Detection (FCD) thresholds, combined with product-specific peptide libraries to properly filter out method-induced artifacts [9,11].

While several works have been published comparing manual and automated sample preparation for TAQ, the performance of NPD across these automated platforms has not been previously reported in the literature [15,19,20,21,22,26]. Without a validated, purity-indicating NPD method that performs reliably to detect unexpected impurities, MAM cannot effectively replace conventional control system tests for biotherapeutic products [1,4,5,6,9,24]. An ideal situation requires demonstrating comparable performance of both TAQ and NPD between manual and automated sample preparation workflows.

To address this gap, this manuscript presents a cross-comparison study evaluating the performance of conventional manual peptide mapping versus an automated Tecan liquid-handling system for both the TAQ and NPD workflows. Building on optimized NPD strategies, this study utilizes a statistically derived FCD threshold alongside a product-specific peptide library and multiple bracketing reference comparisons to maximize sensitivity while eliminating false positives. Comparability for TAQ was first evaluated using an mAb reference standard, namely mAb1. Subsequently, NPD was assessed by spiking a secondary monoclonal antibody (mAb2) into the reference standard mAb1 to simulate unexpected process-related impurities; mAb1 and mAb2 share significant sequence homology, with nine unique tryptic peptides from the digest of mAb2 representing new peaks from mAb1. Ultimately, this study aims to demonstrate that automated sample preparation yields NPD specificity and sensitivity highly comparable to those obtained using manual methods, establishing an essential framework for implementing fully automated MAM workflows for product quality monitoring.

## 2. Results

### 2.1. Overview of MAM TAQ and NPD Comparison Study

In this study, automated sample preparation was done using a Tecan automated liquid handling system and followed nearly identical reduction, alkylation, and digestion reaction conditions as the manual sample preparation workflow. To simulate process-related impurities, mAb2 was spiked into the mAb1 reference standard (RS) at 0.5%, 1%, and 2% *w*/*w* relative abundance across three independent days. For sample preparation, the manual workflow was done by analyst A for Days 1 and 2 while Day 3 was done by analyst B. The automated workflow was done by analyst C across three independent days. For data acquisition, the manual and automated samples were analyzed using separate but identical reverse-phase columns and LC-MS instruments.

For TAQ, the results of 28 product quality attributes (PQAs) of the mAb1 reference standard were evaluated from three independent sample preparations on each day. The attributes included five oxidation sites, three deamidation sites, two succinimide sites, one N-terminal pyroGlu, two glycation sites, one VHS extension, one C-terminal lysine, one proline amidation, one N-clipped peptide, and 11 N-glycoforms.

**Figure 1:**
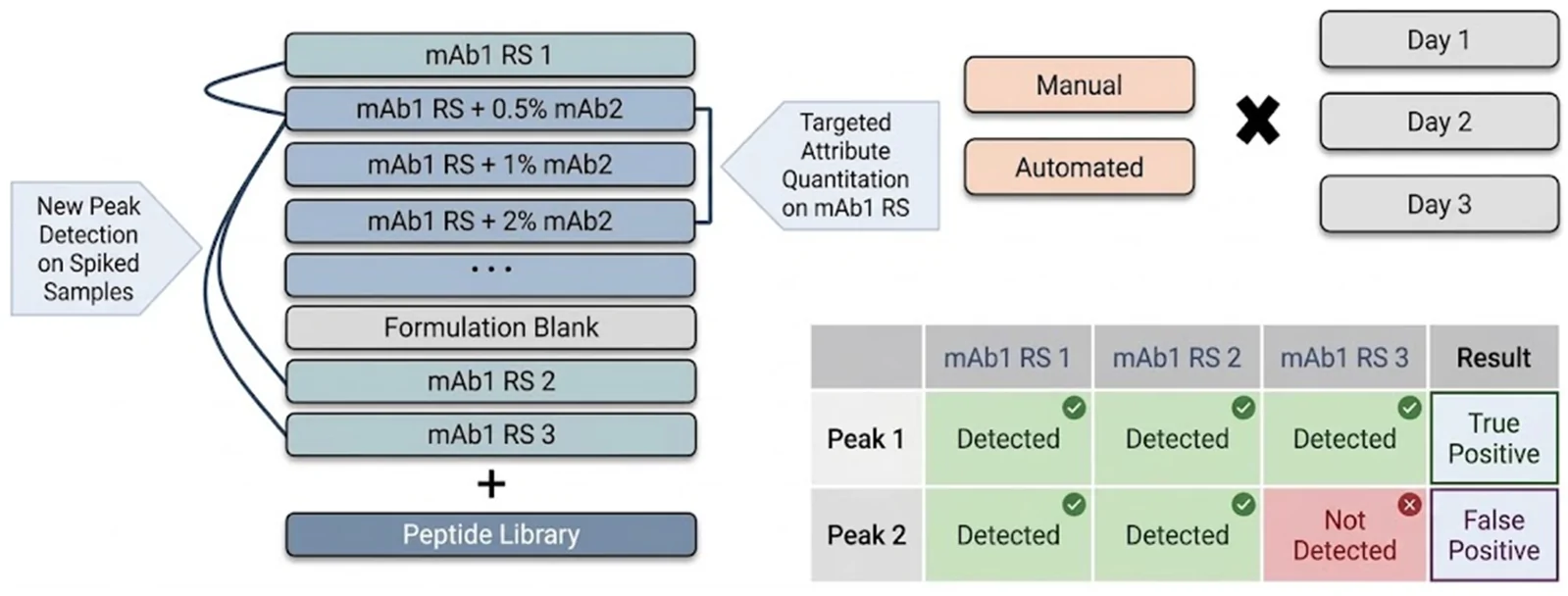
Overview of MAM TAQ and NPD comparison study for manual vs. automated sample preparation workflows. Blue lines represent the three comparisons of spike-in samples with unspiked mAb1 RS.

For NPD, data analysis was adapted from previously described steps [27]. In this workflow, process samples are compared against bracketing mAb1 RS with the addition of a well-characterized product-specific peptide library containing sequence, molecular weight, charge state, and retention time information. For a new peak to be considered a true positive it must be observed in three binary comparisons with the bracketing mAb1 RS (Figure 1).

### 2.2. Comparison of Manual and Automated Sample Preparation Workflows for MAM TAQ

**Figure 2:**
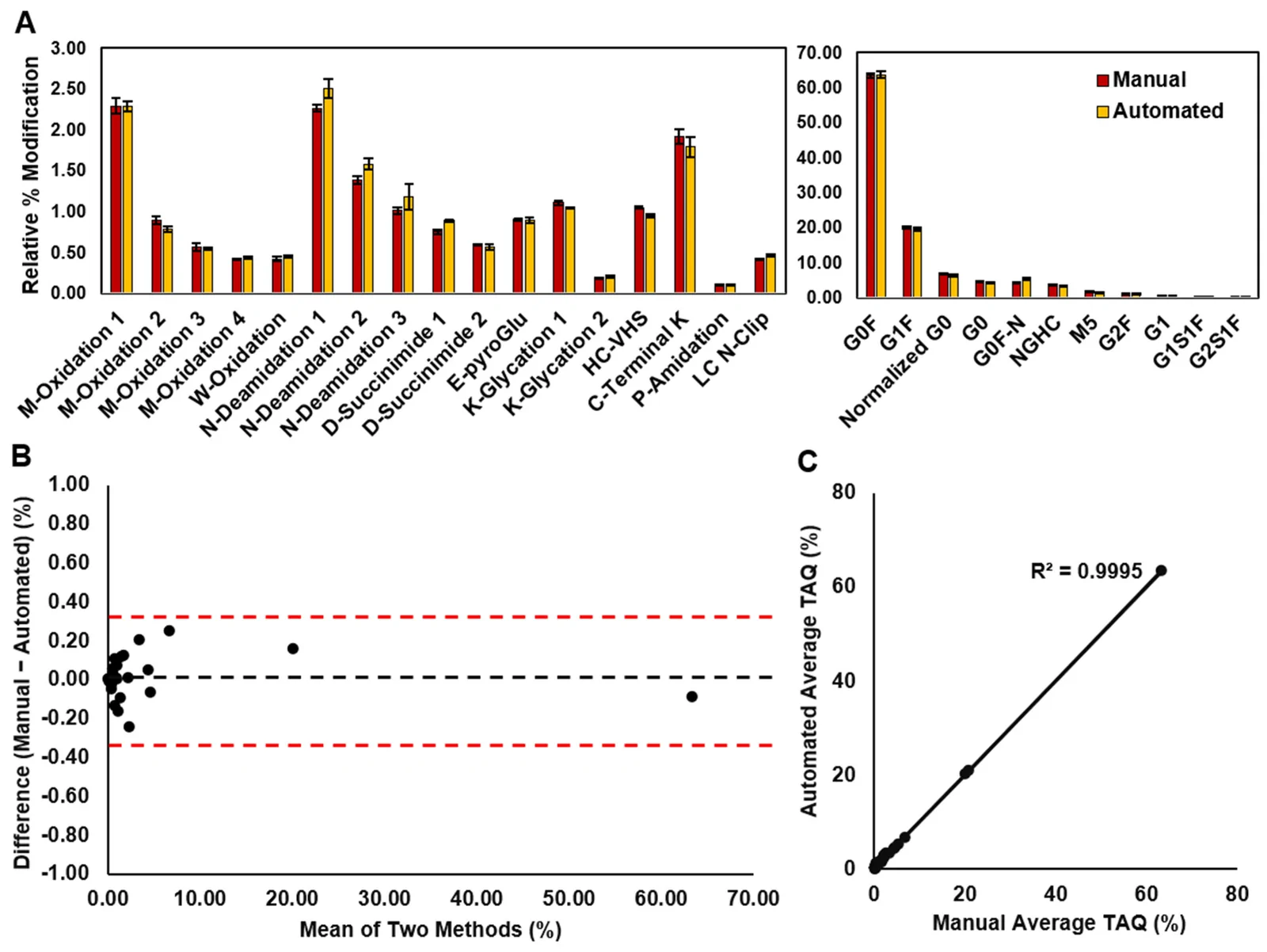
Day 1 comparison of MAM TAQ for mAb1 RS across preparation workflows (*n* = 3 per PQA). (**A**). Average relative % modification of mAb1 PQAs prepared via manual (red) and automated (orange) workflows. Error bars represent +/− 1 SD. (**B**). Bland–Altman analysis comparing manual and automated workflows. The black dashed line represents the mean difference (0.01%), and the red dashed lines represent upper and lower limits of agreement (0.33% and −0.31%). (**C**). Linear regression analysis of average % TAQ of each PQA between manual and automated workflows.

The comparability of the two sample preparation workflows for TAQ was first evaluated using the average TAQ results of the 28 PQAs from three independent mAb1 sample preparations. Figure 2A and Figures S1A and S2A show that the average relative percent modification for all mAb1 PQAs was highly consistent between the manual and automated workflows on Day 1, Day 2, and Day 3, respectively. To further evaluate method agreement and assess potential systematic bias, direct method-comparison analyses were performed. Bland–Altman difference plots showed that the absolute differences between the manual and automated TAQ results were minimal, with all data points falling within upper and lower limits of agreement (Figure 2B and Figures S1B and S2B). Ultimately these plots demonstrate the absence of systematic bias and that the automated method neither consistently over-reported nor under-reported modifications relative to the manual method across the entire dynamic range of evaluated PQAs. Furthermore, linear regression analysis (Figure 2C and Figures S1C and S2C) comparing the average TAQ values of each PQA between the two methods demonstrated excellent linear agreement (*R*^2^ > 0.999), thereby further supporting that the automated liquid handling system yielded highly similar quantitative results and negligible systematic bias relative to the conventional manual workflow.

Next, the intra-day precision, which assesses the level of variability based on sample replicates within the same preparation, was evaluated for each workflow using the average and % coefficient of variation for each modification. All attributes had CVs of less than 15% for both workflows across all three days (Table S1). To directly compare the two sample preparation workflows quantitatively, the inter-day precision, % relative variance, and intermediate precision between the two methods were evaluated. As shown in Table 1, inter-day precision was determined by the average and % CV from three independent mAb1 sample preparations for each day (*n* = 9). Overall, all evaluated PQAs had an inter-day % CV of less than 15%, demonstrating comparable day-to-day reproducibility for both workflows. Furthermore, the percent relative variance of inter-day precision data was calculated from the absolute change in average relative abundance between automated and manual workflows divided by the average relative abundance in the manual workflow (Table 1). Out of the 28 PQAs monitored, all were less than 15% with only three showing a relative variance greater than 10%: M-Oxidation-4 (12.0%), N-Deamidation-2 (12.1%), and C-Terminal K (11.3%). The majority of all other PQAs demonstrated a relative variance of less than 5%. Finally, intermediate precision between the two workflows was assessed by pooling the manual and automated datasets for each PQA evaluated (*n* = 18). The % CV was less than 10% for 24 out of the 28 evaluated PQAs. The few attributes whose % CV was greater than 10% were primarily driven by the variability already present in their respective workflow’s day-to-day variability and not from differences between the two workflows. Overall, the data presented demonstrates minimal quantitative differences, indicating comparable performance between the automated and conventional manual sample preparation workflows.

### 2.3. Comparison of Manual and Automated Sample Preparation Workflows for NPD

To compare manual and automated workflows for NPD, intact mAb2 was spiked into mAb1 at 0.5%, 1%, and 2% w/w relative abundance totaling three independent sample preparations at each level over three days for each sample preparation workflow. Because mAb1 and mAb2 shared significant sequence homology, the digest of mAb2 should yield nine unique tryptic peptides with a diverse set of physiochemical properties, amino acid lengths, charge states and retention times, making them representative of new peaks (Table 2).

For NPD within the PMI software v5.6, Feature Finder parameters were set to extract all potential new peaks without threshold restrictions (absolute minimum intensity = 0 and minimum fold change = 0). To exclude non-peptide species, the minimum isotope correlation was set to 0.95, and the minimum isotope count was set to 3. Spiked mAb1 samples were then compared simultaneously against three bracketing unspiked mAb1 RS samples with a product-specific peptide library to extract new peaks and to eliminate false positives. True new peaks were further isolated from new peaks extracted from baseline noise or signal variance by applying a statistically derived FCD threshold of 3 (see Section 5 and Table S2). Using this workflow, all nine mAb2 peptides were detected at each spike-in level for all sample preparations (Table 3 and Table S3), and no false positives were observed (Table S4). Therefore, the lowest successfully tested level is 0.5% for both manual and automated sample preparation workflows under the conditions tested.

### 2.4. NPD Specificity

**Figure 3:**
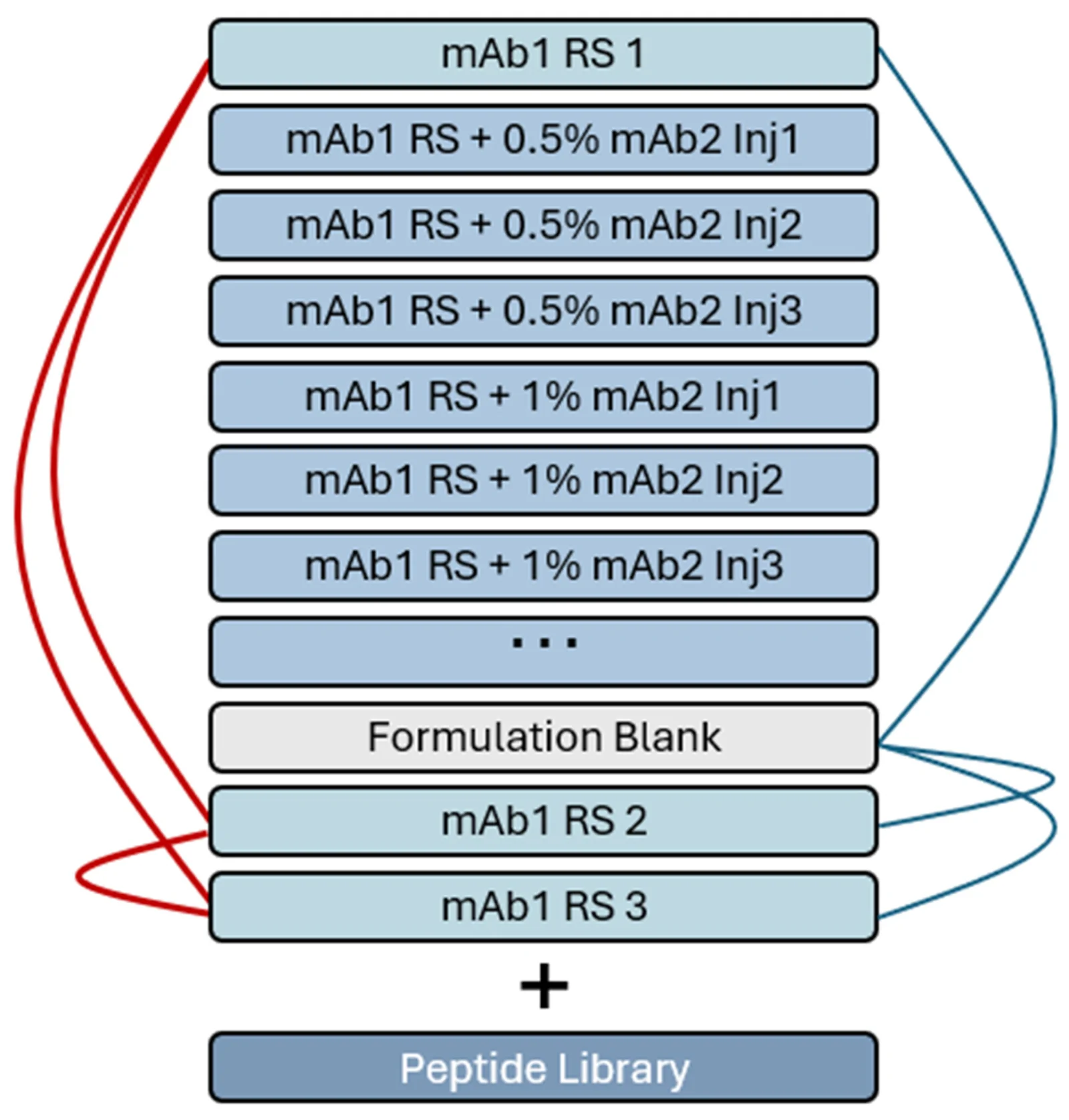
NPD comparisons to determine specificity by comparisons between bracketing mAb1 RS (red lines) and comparisons of formulation blank samples against bracketing mAb1 RS (blue lines).

Assay specificity was determined by the lack of new peaks detected when comparing formulation buffer blank injections with three bracketing mAb1 RS injections and when comparing the bracketing mAb1 RS with each other (Figure 3). Here, any new peaks observed would be introduced during sample preparation and data acquisition. No new peaks were observed in any formulation blank samples or in comparisons between bracketing RS across all workflow days. Thus, NPD was considered qualified for the manual and automated sample preparation workflows with respect to specificity.

## 3. Discussion

In this study, we present the comparison of manual and automated sample preparation for both TAQ and NPD of MAM workflows. Our results show comparable performance for TAQ, which is consistent with the observation from previous studies [15,19,20,21,22]. The Bland–Altman analysis supported a lack of systematic bias between the manual and automated workflows, with all absolute differences being within the upper and lower limits of agreement for all three days. Further linear regression analysis showed close agreement (*R*^2^ > 0.999) between the average % TAQ of each PQA obtained using the two workflows. Both intra-day and inter-day precision remained largely within 15% CV and % relative variance was predominantly below 5%. Pooled intermediate precision between the two workflows also supported comparable performance with all CVs being less than 15%; the few CVs slightly above 10% were driven by day-to-day variability present in both workflows. Ultimately, the data presented herein demonstrates the comparable performance between the automated and manual sample preparation workflows for TAQ.

While our study demonstrates highly comparable performance between the manual and automated workflows, it is important to acknowledge the confounding variables present in the experimental design. Specifically, the manual and automated samples were prepared by different operators (Analysts A and B for the manual workflow; Analyst C for the automated workflow) and were analyzed using separate, albeit identical, LC-MS instruments and reverse-phase columns. This experimental design represents a worst-case scenario typical of a high-throughput laboratory where assays are routinely executed across multiple instruments, columns, and analysts. Consequently, the variability observed in this study encompasses not only the differences between manual and automated sample preparation but also day, analyst, column, and instrument-specific effects. Because the total combined variance of all these factors remained largely within 15% CV and no systematic bias was observed, the data suggests the automated workflow remains highly robust even under these confounding variables. However, we acknowledge that this design limits our ability to definitively isolate the variance originating solely from the sample preparation step itself. Future characterizations utilizing a fully randomized design on a single instrument, or employing a mixed-effects statistical model, would be beneficial.

As the previous literature has pointed out, evaluating performance for NPD is as important as for TAQ to fully implement MAM capabilities with an automated sample preparation workflow [1,5,6,9,24]. NPD is an untargeted differential analysis designed to flag unexpected impurities or changes to the product quality profile of the mAb reference standard. For NPD, implementing it for an automated workflow for MAM requires demonstrating specificity and sensitivity. NPD, however, presents challenges primarily through the generation of false positives. If thresholds are set too low or high, there is the risk of introducing false positives and false negatives, respectively. The previous literature has also emphasized that arbitrary software thresholds are insufficient and that parameters such as the FCD threshold must be empirically derived using statistical approaches [6,9,10]. With these in mind, our NPD workflow utilizes a combination of these approaches to maximize sensitivity while effectively filtering out false positives.

While previous NPD workflows may define thresholds prior to extraction, our approach maximizes sensitivity by extracting peptide-like features without prior filtering [9,12]. Doing this alone will ultimately return excessive false positives. To address this, false positives were filtered out if they met any of the following rejection criteria: (1) they were observed in the product-specific peptide library; (2) they failed to coexist in three reference comparisons with mAb1 RS; or (3) they fell below the statistically derived FCD threshold post-extraction. Here the peptide library contains information pertaining to known peptides, molecular weights, retention times, etc., that can be used for feature matching. If a new peak is observed by the software, it can be immediately cross-referenced with the peptide library and be identified as a false positive. In this case, false positives would typically be known method-induced artifacts such as miscleavages, adducts, or in-source fragments that would have been previously identified during method development [1,10,26]. If a new peak was not previously documented in the peptide library, the new peak can effectively be filtered out if it is not observed in multiple comparisons with RS. While previous workflows have relied on one or two reference comparisons for NPD, increasing the number of binary comparisons has been shown to reduce false positives when considering the increased statistical power of more coexisting comparisons [10,23,27].

Finally, FCD threshold filtering was done post-extraction to address new peaks due to intensity changes. While the peptide library and bracketing comparisons filter out false positives associated with sample preparation-related artifacts, FCD thresholding serves to filter out false positives associated with variability of the MS signal [10,12,26]. Here, instead of arbitrary selection, the FCD threshold was statistically derived when considering the worst-case MS signal variability of each peptide. Although the calculated FCD threshold differs between days and between the two workflows, the likelihood of false negatives in this workflow is low because the initial NPD FCD threshold was set to 0, ensuring that all peptide-like features were extracted before the FCD threshold of 3 was applied as a post-extraction filter.

However, it is important to acknowledge that this NPD performance was evaluated using a single impurity model. While the nine unique mAb2 tryptic peptides encompass a diverse range of lengths, charge states, and retention times, this model does not encompass all possible impurity profiles. Specifically, this study relied on unmodified, wildtype peptides from mAb2 to simulate new peaks, rather than evaluating PQAs present at very low relative abundances (e.g., <0.5%). NPD sensitivity and overall performance may differ for low-ionization peptides, very hydrophilic or hydrophobic species, unexpected chemical modifications, sequence variants, host–cell proteins, or other unexpected impurities such as leachables. Future evaluations incorporating a broader array of impurity classes would be beneficial to define the sensitivity and specificity of NPD for the automated workflow. Ultimately for this study, under the conditions tested, all mAb2 peptides at each level were identified and, more importantly, no false positives were observed in any of the datasets, especially at the lowest level tested of 0.5%.

## 4. Materials and Methods

### 4.1. Materials

Iodoacetic acid (IAA), 8M guanidine hydrochloride (Gn-HCl), trifluoroacetic acid (TFA), and LC-MS-grade water were from Thermo Fisher (Sunnyvale, CA, USA). Tris base, calcium chloride, 1N sodium hydroxide, LC-MS grade mobile phase A 0.1% formic acid (FA) in water, and LC-MS grade mobile phase B 0.1% FA in acetonitrile were from J.T. Baker (Philipsburg, NJ, USA). Dithiothreitol (DTT), L-methionine, and sequencing-grade modified trypsin were from Promega (Madison, WI, USA); Ethylenediaminetetraacetic acid (EDTA) was from Sigma (St. Louis, MO, USA). NAP-5 columns were Sephadex G-25 DNA grade and from Cytiva (Marlborough, MA, USA).

Two IgG1 monoclonal antibodies, mAb1 and mAb2, manufactured at Genentech (South San Francisco, CA, USA) were used in this study. mAb1 was used as the reference standard while mAb2 was serially diluted and spiked into mAb1 to create 0.5%, 1%, and 2% *w*/*w* relative abundance spike-in samples.

### 4.2. Manual Sample Preparation

For manual sample preparation, 250 µg of sample was diluted in 6 M Gn-HCl, 360 mM tris, and 2 mM EDTA at pH 7.0 and reduced with DTT at a 16 mM final concentration for 1 h at 37 °C. After cooling to room temperature, the samples were alkylated with IAA at a 39 mM final concentration and incubated in the dark for 15 min at room temperature. The alkylation reaction was quenched by adding DTT at a 8 mM final concentration and then was buffer exchanged using NAP-5 columns into digestion buffer (50 mM tris, 2 mM calcium chloride, pH 7.5). Prior to buffer exchange, the NAP-5 columns were equilibrated with 10 mL of the digestion buffer. The samples were then digested by trypsin at a 1:16 *w*/*w* enzyme-to-protein ratio for 1 h at 37 °C. L-methionine and TFA were added to the digested sample to make final concentrations of 20 mM and 0.2% *v*/*v*, respectively. The samples were then analyzed by LC-MS or stored at −70 °C until further analysis.

### 4.3. Automated Sample Preparation

Automated sample preparation was done using a TECAN Fluent 1080 liquid handling system (Tecan Group Ltd., Männedorf, Switzerland). The Fluent 1080 was integrated with an Inheco ODTC 96 Thermal Cycler (INHECO GmbH, Planegg, Germany) for incubations and a BioNex HiG 3 Centrifuge (San Jose, CA, USA). The system’s 8-Channel Flexible Channel Arm and Robotic Gripper Arm were used for all liquid transfers and plate movements. The automated workflow used 96-well PCR plates from Bio-Rad (Hercules, CA, USA) for sample loading as well as 96-well deep well plates and 50 mL plates from Axygen (Union City, CA, USA) for liquid transfers and buffer storage, respectively. Final concentrations for reduction, alkylation, digestion, and quenching were identical to the manual workflow.

### 4.4. LC-MS/MS Analysis

Around 6 µg of sample was analyzed with LC-MS/MS analysis using a Thermo Vanquish UHPLC system connected to an Orbitrap Thermo Q Exactive Plus mass spectrometer (Thermo Fisher Scientific, Sunnyvale, CA, USA) equipped with a heated electrospray ionization source. Reverse-phase separation was performed on a Waters Acquity Premier Peptide CSH C18 column 130 Å, 1.7 μm, 2.1 mm × 150 mm (Milford, MA, USA) at 60 °C and a 0.20 mL/min flow rate. Mobile phase A was 0.1% FA in water, and mobile phase B was 0.1% FA in acetonitrile. The gradient started at 0% mobile phase B for 2 min and then increased to 1% B at 3 min, 13% B at 8 min, 35% B for 43 min, and finally to 95% B at 45 min. The column was then washed with three alternating cycles between 1% and 95% B before being equilibrated with 0% B for 18 min.

The experimental data were acquired in the positive mode using a Top 8 data-dependent data acquisition method with heated electrospray ion source settings of 3.5 kV spray voltage, 250 °C capillary temperature, 250 °C auxiliary gas heater temperature, 55 RF S-Lens, sheath gas flow rate of 30, auxiliary gas flow rate of 6, and sweep gas flow rate of 0. Full MS scans were performed at 70 K resolution, an AGC target of 1e6 a max injection time of 100 ms, and a scan range from 200 to 2000 *m*/*z* while MS2 scans were performed at 17.5 K resolution, an AGC target of 1e5, a max injection time of 50 ms, and an isolation window of 2.0 *m*/*z*.

### 4.5. Data Analysis for TAQ and NPD

For targeted attribute quantitation, a peptide workbook containing 28 PQAs for the mAb1 reference standard was generated using BioPharma Finder (Thermo Scientific, Waltham, MA, USA) and filtered to contain peptides with up to one miscleavage. The peptide workbook was then imported into Chromeleon v7.2.10 software (Thermo Scientific) to generate a processing method. Targeted attribute quantitation was performed using the ICIS detection algorithm, a precursor mass tolerance of 10 ppm, an isotope dot product ≥0.9, and peak and apex alignment ≤0.5 min. Relative quantitation of modifications was calculated by dividing the peak area of the modified peptide by the total peak area of the modified and wildtype peptides.

### 4.6. NPD Data Analysis

NPD was adapted from previously described steps in Cao et al., 2025 [27]. In brief, process samples are compared against bracketing mAb1 reference standards using a product-specific peptide library (containing sequence, molecular weight, charge, and retention time data). To be confirmed as a true positive, a new peak must be observed in three binary comparisons with the bracketing standards. For new peak detection using Protein Metrics Byos v5.6 (Cupertino, CA, USA), a custom workflow was made to allow for three simultaneous comparisons along with a product-specific peptide library for feature matching. Feature finder was enabled with a minimum isotope correlation of 0.95, mass range of 500–8000, maximum feature count of 50, minimum fold change of 0, absolute minimum intensity of 0, and minimum isotope count of 3. An FCD threshold of 3 was then applied after peak extraction.

### 4.7. Calculation of FCD Threshold

For a statistical determination of the FCD threshold, a fold change value was calculated for each wildtype peptide from bracketing mAb1 RS samples by dividing the peptide intensity by the lowest intensity across the injection sequence for that peptide. To define the upper statistical limit of intensity variability, a calculated FCD threshold for each injection was determined using u + 3σ where u is the average fold change and σ is the standard deviation. Since this value represents the upper limit of signal variance, the defined FCD threshold was conservatively set to exceed this. Across the mAb1 RS injections, the calculated FCD threshold ranged from 1.8 to 2.4. Consequently, the defined FCD threshold was set to 3 to safely exceed the worst-case fold change in the most variable peptides across both sample preparation workflows and therefore filter out false positives attributed to inherent signal variance (Table S2).

## 5. Conclusions

To fully implement automated MAM workflows, similar NPD performance regarding specificity and sensitivity compared to manual workflows needs to be demonstrated [26,28,29]. In this study, we have demonstrated that automated sample preparation workflows enable high-throughput monitoring of targeted attributes with great reproducibility, showing similar performance and minimal systematic bias compared to the manual workflow, which is consistent with previous studies [19,20,21,22]. Furthermore, our study demonstrated similar NPD sensitivity and specificity between automated and manual workflows. To the best of our knowledge, our work is the first study that shows comparable performance of NPD between automated and manual sample preparation workflows. Ultimately, this work provides an essential framework for fully implementing MAM workflows with both TAQ and NPD using automated liquid handling systems.

## Figures and Tables

**Table 1:** Inter-day precision, % relative variance, and intermediate precision of manual versus automated sample preparation for the 28 PQAs evaluated for TAQ for mAb1.

Modification	Inter-Day Precision (*n* = 9) *				% Relative Variance **	Intermediate Precision (*n* = 18) ***	
	Average Relative Abundance		% CV				
	Manual	Automated	Manual	Automated		Average Relative Abundance	% CV
M-Oxidation 1	2.4	2.4	3.6	4.2	0.6	2.4	3.8
M-Oxidation 2	0.9	0.8	6.2	4.7	3.3	0.8	5.6
M-Oxidation 3	0.5	0.6	9.8	7.7	3.8	0.5	8.8
M-Oxidation 4	0.4	0.5	7.6	5.6	12.0	0.4	8.6
W-Oxidation	0.4	0.4	13.6	14.2	6.6	0.4	13.5
N-Deamidation 1	2.2	2.4	3.3	6.8	6.3	2.3	6.1
N-Deamidation 2	1.3	1.5	4.8	6.9	12.1	1.4	8.3
N-Deamidation 3	1.1	1.1	11.3	9.0	5.3	1.1	10.2
D-Succinimide 1	0.8	0.9	10.1	4.9	8.5	0.9	8.6
D-Succinimide 2	0.6	0.7	6.9	11.1	2.9	0.7	11.3
E-pyroGlu	1.0	0.9	7.9	2.6	4.6	0.9	6.3
K-Glycation 1	1.1	1.1	4.7	3.6	2.0	1.1	4.2
K-Glycation 2	0.2	0.2	2.7	6.9	1.4	0.2	5.1
HC-VHS	1.0	1.0	5.7	7.7	2.9	1.0	6.7
C-Terminal K	1.9	1.7	4.0	9.5	11.3	1.8	9.1
P-Amidation	0.1	0.1	2.7	5.3	0.3	0.1	4.1
LC N-Clip	0.5	0.5	12.6	6.9	2.2	0.5	9.9
G0F	63.3	64.4	0.5	2.1	1.8	63.9	1.8
G1F	20.1	19.5	0.8	3.6	3.3	19.8	3.0
Normalized G0	6.8	6.3	2.2	4.9	6.6	6.5	5.1
G0	4.6	4.3	2.2	3.9	5.5	4.5	4.2
G0F-N	4.4	4.5	8.0	6.2	2.6	4.4	11.4
NGHC	3.5	3.4	2.0	2.6	3.4	3.5	2.9
M5	1.7	1.6	4.9	4.2	4.9	1.6	5.1
G2F	1.1	1.0	4.2	9.2	8.0	1.0	8.0
G1	0.6	0.6	3.3	9.3	2.5	0.6	6.8
G1S1F	0.3	0.3	4.3	5.3	0.8	0.3	4.7
G2S1F	0.2	0.2	3.6	2.2	5.8	0.2	6.6

* Inter-day precision calculated from 3 independent sample preparations for each day (*n* = 9). ** % Relative Variance calculated from absolute value (Automated − Manual)/(Manual) × 100% from inter-day precision average relative abundance data. *** Intermediate precision calculated from manual and automated data pooled (*n* = 18, 3 independent sample preparations per workflow per day).

**Table 2:** Overview of unique mAb2 peptides used for NPD evaluation.

mAb2 Peptide	HC or LC	Length (AA)	Charge State	Retention Time (min)
Peptide-1	LC	7	2	8.0–8.2
Peptide-2	LC	6	2	8.6–9.2
Peptide-3	HC	7	2	11.1–11.7
Peptide-4	LC	9	2	13.0–13.2
Peptide-5	LC	18	2, 3	13.0–13.9
Peptide-6	HC	20	2, 3	19.9–20.2
Peptide-7	HC	19	2, 3	29.9–30.3
Peptide-8	HC	25	2, 3	31.4–32.7
Peptide-9	LC	42	3, 4	37.3–38.0

**Table 3:** Summary of NPD for mAb2 peptides between manual and automated workflows.

Parameter	Criteria	% Spike	Manual	Automated
Number of Unique mAb2 Peptides (expected 9)	≥1 Peptide detected	0.5%	9 Peptides for Days 1–3	
		1%	9 Peptides for Days 1–3	
		2%	9 Peptides for Days 1–3	

## Data Availability

The original contributions presented in this study are included in the article/Supplementary Materials. Further inquiries can be directed to the corresponding author.

## Supplementary Materials

The following supporting information can be downloaded at: https://www.mdpi.com/article/10.3390/ph19101605/s1, Figure S1: Day 2 comparison of MAM TAQ for mAb1 RS across preparation workflows; Figure S2: Day 3 comparison of MAM TAQ for mAb1 RS across preparation workflows; Table S1: Intra-day precision shown as % CV of manual versus automated sample preparation for the 28 mAb1 PQAs evaluated for TAQ; Table S2: Calculation of FCD Threshold; Table S3: Extracted-Ion Chromatogram (EIC) Area of each mAb2 peptide for 0.5% spike level sample between Manual and Automated workflows; Table S4: NPD workflow performance of new peaks after each filter step for 0.5% mAb2 spike level samples.

## Abbreviations

MAM	Multi-Attribute Method
NPD	New Peak Detection
LC-MS	Liquid Chromatography Mass Spectrometry
mAb	Monoclonal Antibody
PQA	Product Quality Attribute
TAQ	Targeted Attribute Quantitation
CV	Coefficient of Variation
QC	Quality Control
FCD	Fold Change Detection
