# Supplementary material for: Qualification of Targeted Attribute Quantitation and New Peak Detection for Automated Multi-Attribute Method Workflows Using Liquid Handling Systems
Source: Pharmaceuticals (Basel). 2026 Oct 9;19(10):1605. doi: 10.3390/ph19101605 (PMC13708717; doi:10.3390/ph19101605)
Supplement: Supplementary file 1 [file pharmaceuticals-19-01605-s001.zip › pharmaceuticals-4559246-supplementary.pdf]

# Supplemental

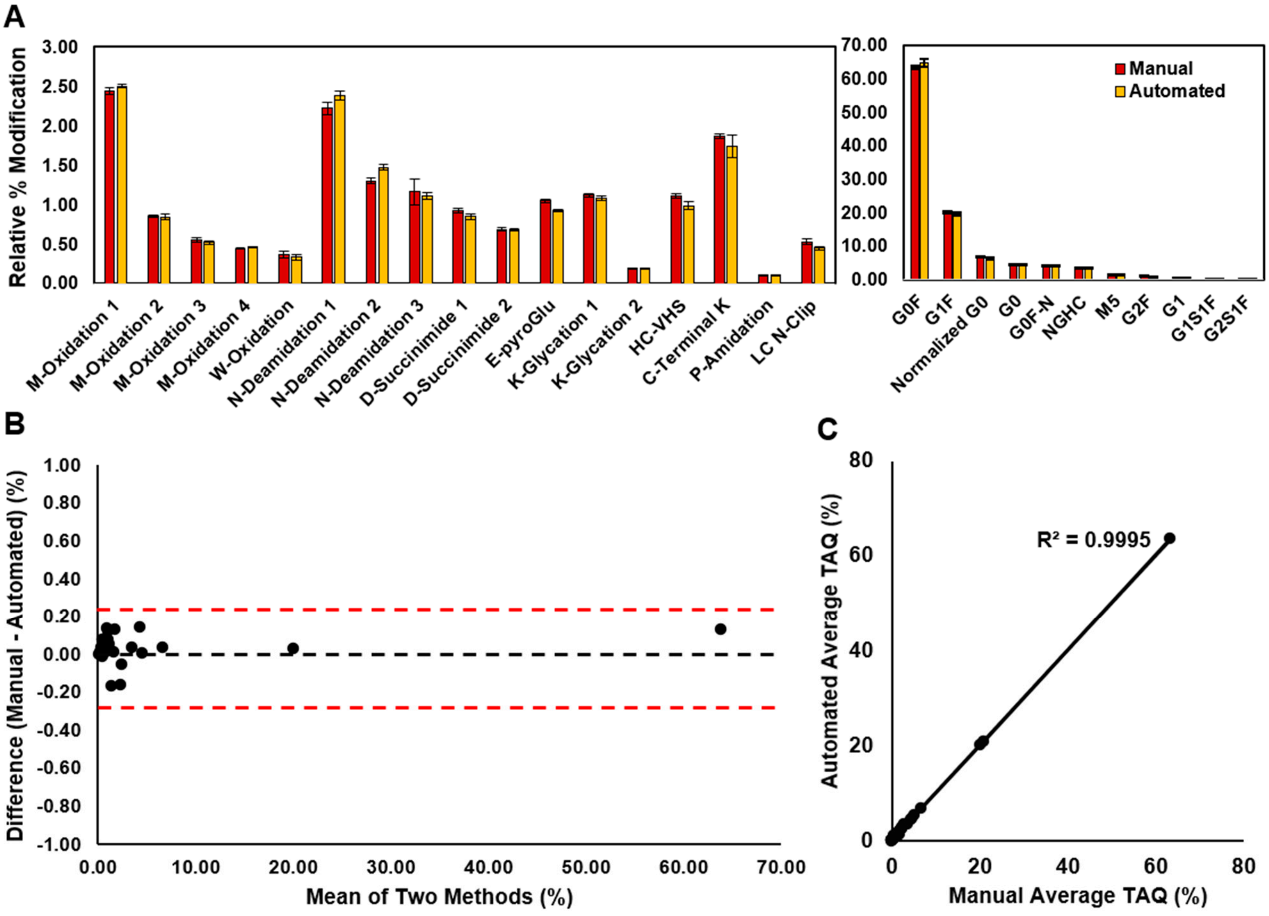


**Figure S1.** Day 2 comparison of MAM TAQ for mAb1 RS across preparation workflows (n=3 per PQA). A). Average relative % modification of mAb1 PQAs prepared via Manual (red) and Automated (orange) workflows. Error bars represent +/- 1 SD. B). Bland-Altman analysis comparing Manual and Automated workflows. The black dashed line represents the mean difference (0.01%) and the red dashed lines represent upper and lower limits of agreement (0.25% and -0.25%). C). Linear regression analysis of average % TAQ of each PQA between Manual and Automated workflows.

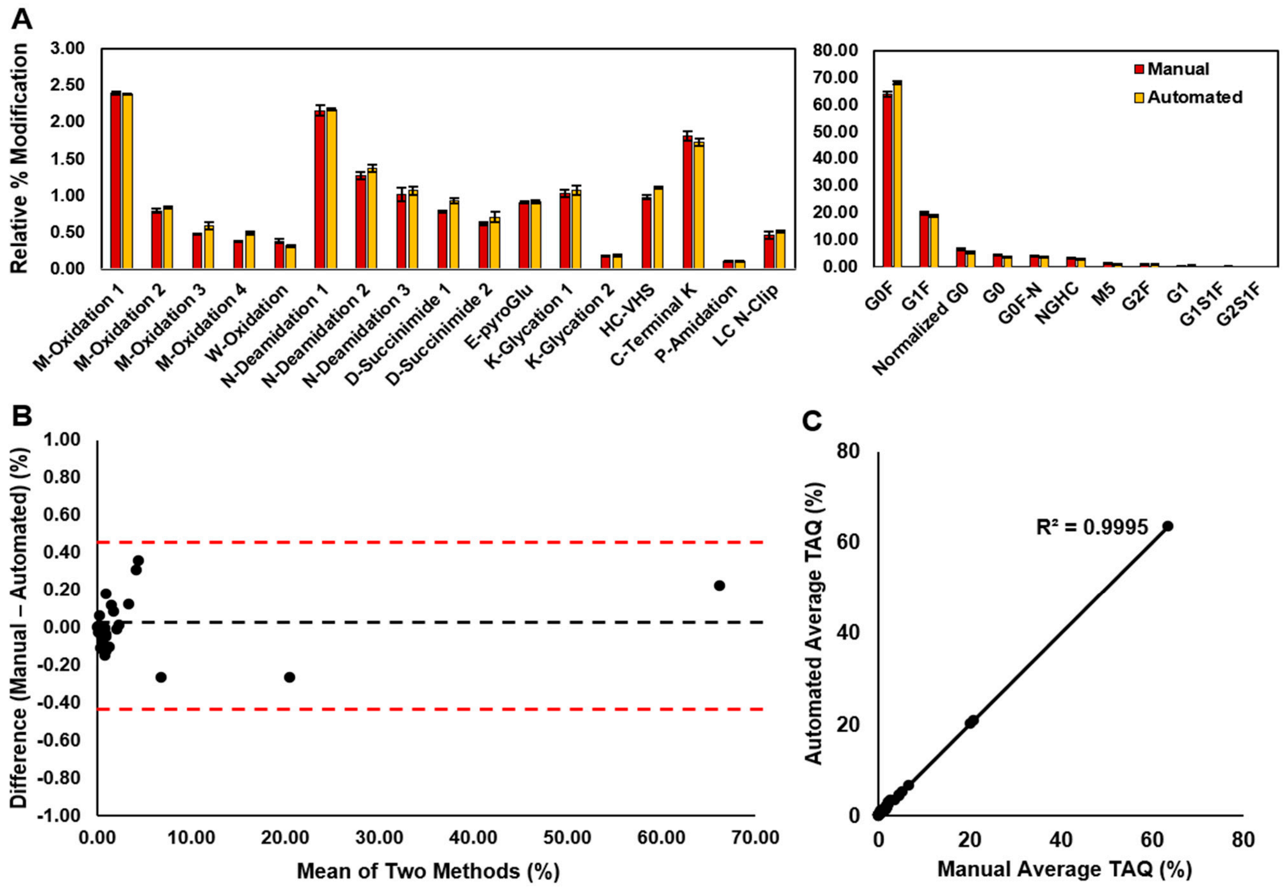


**Figure S2.** Day 3 comparison of MAM TAQ for mAb1 RS across preparation workflows (n=3 per PQA). A). Average relative % modification of mAb1 PQAs prepared via Manual (red) and Automated (orange) workflows. Error bars represent +/- 1 SD. B). Bland-Altman analysis comparing Manual and Automated workflows. The black dashed line represents the mean difference (0.01%) and the red dashed lines represent upper and lower limits of agreement (0.45% and -0.43%). C). Linear regression analysis of average % TAQ of each PQA between Manual and Automated workflows.

**Table S1.** Intra-day precision shown as % CV of manual versus automated sample preparation for the 28 mAb1 PQAs evaluated for TAQ. % CV calculated from 3 independent sample preparations for each day (n=3).

| | Day 1 | | Day 2 | | Day 3 | |
|---|---|---|---|---|---|---|
| **Modification** | **Manual** | **Automated** | **Manual** | **Automated** | **Manual** | **Automated** |
| M-Oxidation 1 | 4.21 | 2.49 | 1.70 | 0.86 | 0.78 | 0.17 |
| M-Oxidation 2 | 5.55 | 4.57 | 1.66 | 4.26 | 3.49 | 2.30 |
| M-Oxidation 3 | 9.32 | 3.05 | 5.41 | 3.15 | 1.04 | 8.16 |
| M-Oxidation 4 | 1.11 | 2.32 | 1.64 | 2.29 | 1.94 | 4.90 |
| W-Oxidation | 7.70 | 3.42 | 10.42 | 10.93 | 6.68 | 4.88 |
| N-Deamidation 1 | 1.74 | 4.62 | 3.63 | 2.70 | 3.34 | 0.77 |
| N-Deamidation 2 | 3.33 | 4.08 | 2.85 | 2.27 | 3.98 | 3.64 |
| N-Deamidation 3 | 4.30 | 3.67 | 3.72 | 3.59 | 8.85 | 4.96 |
| D-Succinimide 1 | 4.01 | 1.64 | 3.47 | 4.06 | 1.85 | 3.67 |

| | | | | | | |
|---|---|---|---|---|---|---|
| D-Succinimide 2 | 1.05 | 6.24 | 2.71 | 1.99 | 3.69 | 9.69 |
| E-pyroGlu | 1.85 | 3.63 | 2.11 | 1.64 | 2.24 | 2.36 |
| K-Glycation 1 | 2.62 | 0.53 | 2.00 | 2.19 | 4.59 | 5.66 |
| K-Glycation 2 | 2.17 | 5.26 | 3.16 | 1.20 | 0.83 | 5.53 |
| HC-VHS | 0.68 | 2.16 | 2.46 | 5.72 | 2.73 | 1.37 |
| C-Terminal K | 4.64 | 8.00 | 1.70 | 8.14 | 3.50 | 3.19 |
| P-Amidation | 1.63 | 7.46 | 4.41 | 3.72 | 1.48 | 3.40 |
| LC N-Clip | 2.63 | 3.52 | 7.21 | 4.89 | 5.60 | 3.44 |
| G0F | 0.32 | 0.68 | 0.43 | 0.55 | 0.38 | 1.27 |
| G1F | 0.46 | 1.19 | 0.27 | 0.38 | 1.32 | 3.90 |
| Normalized G0 | 1.16 | 2.43 | 2.75 | 1.18 | 2.39 | 2.33 |
| G0 | 0.97 | 2.15 | 2.85 | 1.03 | 2.65 | 1.34 |
| G0F-N | 1.09 | 2.19 | 1.50 | 1.20 | 1.46 | 1.38 |
| NGHC | 1.28 | 2.35 | 2.81 | 2.46 | 1.44 | 1.91 |
| M5 | 5.42 | 1.63 | 6.32 | 1.88 | 2.50 | 6.41 |
| G2F | 0.74 | 3.21 | 6.31 | 9.08 | 2.94 | 12.23 |
| G1 | 5.23 | 8.71 | 0.62 | 7.97 | 2.83 | 8.19 |
| G1S1F | 1.00 | 6.45 | 3.87 | 3.49 | 3.97 | 5.73 |
| G2S1F | 2.49 | 3.95 | 2.59 | 3.01 | 4.31 | 2.82 |

**Table S2.** Calculation of FCD Threshold. The average fold change +/- 3 SD on each day for manual and automated workflows (n=108, 3 injections x 36 mAb1 wildtype peptides).

| Workflow | Day | Average FC | SD | Average FC + 3SD |
|---|---|---|---|---|
| Manual | 1 | 1.2 | 0.2 | 1.8 |
| | 2 | 1.2 | 0.2 | 1.8 |
| | 3 | 1.4 | 0.3 | 2.2 |
| Automated | 1 | 1.4 | 0.3 | 2.4 |
| | 2 | 1.4 | 0.3 | 2.2 |
| | 3 | 1.3 | 0.3 | 2.1 |

**Table S3.** Extracted-Ion Chromatogram (EIC) Area of each mAb2 peptide for 0.5% spike-level sample between Manual and Automated workflows. % CV calculated from 3 independent sample preparations for each day (n=3) and Inter-Day % CV calculated from 1 sample preparation for each day (n=3).

| Workflow | mAb2 Peptide | Replicate | Day 1 | | Day 2 | | Day 3 | | Inter-Day % CV |
|---|---|---|---|---|---|---|---|---|---|
| | | | EIC Area | % CV | EIC Area | % CV | EIC Area | % CV | |
| **Manual** | Peptide 1 | 1 | 4.2E+06 | 3.1 | 4.15E+06 | 11.7 | 4.2E+06 | 3.1 | 0.3 |
| | | 2 | 4.2E+06 | | 3.28E+06 | | 4.2E+06 | | 13.6 |
| | | 3 | 4.4E+06 | | 3.72E+06 | | 4.4E+06 | | 9.6 |
| | Peptide 2 | 1 | 6.9E+06 | 10.7 | 6.39E+06 | 10.5 | 6.9E+06 | 10.7 | 4.0 |
| | | 2 | 5.9E+06 | | 5.19E+06 | | 5.9E+06 | | 6.9 |
| | | 3 | 7.3E+06 | | 5.65E+06 | | 7.3E+06 | | 13.7 |
| | Peptide 3 | 1 | 1.7E+07 | 3.9 | 1.50E+07 | 4.9 | 1.7E+07 | 3.9 | 6.0 |
| | | 2 | 1.6E+07 | | 1.40E+07 | | 1.6E+07 | | 6.4 |
| | | 3 | 1.7E+07 | | 1.37E+07 | | 1.7E+07 | | 11.4 |
| | Peptide 4 | 1 | 1.7E+07 | 6.0 | 1.67E+07 | 8.3 | 1.7E+07 | 6.0 | 0.6 |
| | | 2 | 1.5E+07 | | 1.45E+07 | | 1.5E+07 | | 1.6 |

| | | | | | | | | | |
|---|---|---|---|---|---|---|---|---|---|
| | | 3 | 1.6E+07 | | 1.45E+07 | | 1.6E+07 | | 6.2 |
| | | 1 | 1.0E+07 | | 1.06E+07 | | 1.0E+07 | | 1.1 |
| | Peptide 5 | 2 | 9.1E+06 | 7.6 | 9.68E+06 | 5.1 | 9.1E+06 | 7.6 | 3.4 |
| | | 3 | 1.0E+07 | | 9.81E+06 | | 1.0E+07 | | 3.7 |
| | | 1 | 5.8E+06 | | 6.42E+06 | | 5.8E+06 | | 6.2 |
| | Peptide 6 | 2 | 5.3E+06 | 5.8 | 6.21E+06 | 2.9 | 5.3E+06 | 5.8 | 9.3 |
| | | 3 | 6.0E+06 | | 6.07E+06 | | 6.0E+06 | | 1.1 |
| | | 1 | 5.5E+06 | | 4.44E+06 | | 5.5E+06 | | 12.3 |
| | Peptide 7 | 2 | 5.2E+06 | 6.7 | 5.45E+06 | 10.2 | 5.2E+06 | 6.7 | 2.7 |
| | | 3 | 4.8E+06 | | 4.96E+06 | | 4.8E+06 | | 1.4 |
| | | 1 | 1.4E+06 | | 1.38E+06 | | 1.4E+06 | | 0.5 |
| | Peptide 8 | 2 | 1.3E+06 | 5.2 | 1.55E+06 | 6.7 | 1.3E+06 | 5.2 | 12.6 |
| | | 3 | 1.3E+06 | | 1.55E+06 | | 1.3E+06 | | 10.9 |
| | | 1 | 1.4E+06 | | 1.67E+06 | | 1.4E+06 | | 10.3 |
| | Peptide 9 | 2 | 9.6E+05 | 22.2 | 1.41E+06 | 8.5 | 9.6E+05 | 22.2 | 23.3 |
| | | 3 | 9.8E+05 | | 1.50E+06 | | 9.8E+05 | | 26.0 |
| | | 1 | 6.19E+06 | | 4.35E+06 | | 6.19E+06 | | 19.1 |
| | Peptide 1 | 2 | 4.94E+06 | 12.7 | 3.75E+06 | 13.4 | 4.94E+06 | 12.7 | 15.1 |
| | | 3 | 5.08E+06 | | 3.33E+06 | | 5.08E+06 | | 22.4 |
| | | 1 | 8.17E+06 | | 5.81E+06 | | 8.17E+06 | | 18.5 |
| | Peptide 2 | 2 | 7.43E+06 | 4.7 | 5.41E+06 | 5.2 | 7.43E+06 | 4.7 | 17.3 |
| | | 3 | 7.80E+06 | | 5.25E+06 | | 7.80E+06 | | 21.2 |
| | | 1 | 2.18E+07 | | 1.62E+07 | | 2.18E+07 | | 16.1 |
| | Peptide 3 | 2 | 1.87E+07 | 14.9 | 1.48E+07 | 6.4 | 1.87E+07 | 14.9 | 12.9 |
| | | 3 | 1.62E+07 | | 1.44E+07 | | 1.62E+07 | | 6.6 |
| | | 1 | 2.35E+07 | | 1.74E+07 | | 2.35E+07 | | 16.5 |
| | Peptide 4 | 2 | 2.01E+07 | 12.0 | 1.59E+07 | 8.1 | 2.01E+07 | 12.0 | 13.1 |
| | | 3 | 1.87E+07 | | 1.49E+07 | | 1.87E+07 | | 12.8 |
| | | 1 | 1.61E+07 | | 1.24E+07 | | 1.61E+07 | | 14.6 |
| **Automated** | Peptide 5 | 2 | 1.39E+07 | 11.6 | 1.18E+07 | 7.7 | 1.39E+07 | 11.6 | 9.1 |
| | | 3 | 1.29E+07 | | 1.06E+07 | | 1.29E+07 | | 10.6 |
| | | 1 | 9.68E+06 | | 7.53E+06 | | 9.68E+06 | | 13.8 |
| | Peptide 6 | 2 | 8.00E+06 | 13.3 | 6.73E+06 | 8.1 | 8.00E+06 | 13.3 | 9.7 |
| | | 3 | 7.55E+06 | | 6.46E+06 | | 7.55E+06 | | 8.7 |
| | | 1 | 6.76E+06 | | 4.65E+06 | | 6.76E+06 | | 20.1 |
| | Peptide 7 | 2 | 6.21E+06 | 8.1 | 5.22E+06 | 9.1 | 6.21E+06 | 8.1 | 9.7 |
| | | 3 | 5.75E+06 | | 5.58E+06 | | 5.75E+06 | | 1.7 |
| | | 1 | 2.00E+06 | | 2.37E+06 | | 2.00E+06 | | 10.2 |
| | Peptide 8 | 2 | 2.01E+06 | 20.2 | 2.18E+06 | 8.6 | 2.01E+06 | 20.2 | 4.8 |
| | | 3 | 1.37E+06 | | 2.00E+06 | | 1.37E+06 | | 22.8 |
| | | 1 | 1.10E+06 | | 1.22E+06 | | 1.10E+06 | | 6.2 |
| | Peptide 9 | 2 | 9.22E+05 | 9.2 | 1.06E+06 | 16.4 | 9.22E+05 | 9.2 | 8.4 |
| | | 3 | 9.77E+05 | | 8.78E+05 | | 9.77E+05 | | 6.0 |

**Table S4.** NPD workflow performance of new peaks after each filter step for 0.5% mAb2 spike-level samples. Data shows the aggregate number of new peaks for each workflow day (n=3). The expected number of true positive new peaks is 27 per day (9 unique mAb2 peptides per replicate).

| Filter Step | Manual | | | Automated | | |
|---|---|---|---|---|---|---|
| | Day 1 | Day 2 | Day 3 | Day 1 | Day 2 | Day 3 |
| **Total False Positives** | 927 | 831 | 808 | 987 | 834 | 901 |
| **Feature Matching by Peptide Library** | 244 | 301 | 209 | 404 | 540 | 288 |
| **Triplicate Comparisons with mAb1 RS** | 217 | 289 | 188 | 388 | 528 | 260 |
| **FCD Threshold** | 27 | 27 | 27 | 27 | 27 | 27 |
| **Final False positives** | 0 | 0 | 0 | 0 | 0 | 0 |
